# Some Special Aneurysms

**Published:** 1908-05-23

**Authors:** 


### Points in Surgery.

### SOME SPECIAL ANEURYSMS.

The classical symptoms to which an aneurysm gives rise have already been described; but it must be remembered that these will vary widely according to the anatomical relations of the artery involved, and a short description of some of the more prominent phenomena associated with special aneurysms will not be out of place. It may be of the utmost importance even to a surgeon to be able to recognise the presence of an intra-thoracic aneurysm because it may cause dysphagia by pressing on the œsophagus, and to pass an œsophageal tube is a highly dangerous proceeding under these circumstances. Aneurysms of the thoracic aorta may be divided into two classes—(*i*) of the ascending arch, and (*ii*) of the transverse arch. The former of these has been called the aneurysm of physical signs, since it attacks the most superficial part of the vessel and often calls attention to its presence in the first place by causing an obvious pulsating swelling to the outer side of the sterno-mastoid in the base of the neck, while the second is sometimes known as the aneurysm of symptoms, because, being situated deeply, it makes its presence known by the pathological effects of its pressure upon adjacent viscera.

Aneurysm of the ascending arch generally presents as a pulsating tumour behind the right sterno-clavicular articulation, or may come to the surface by eroding the sternum or chest-wall. There is no difference in the radial pulses, since both are modified to the same extent; in this point it differs from an innominate aneurysm, in which the right radial pulse is diminished and delayed. In other respects the two are not dissimilar, an innominate aneurysm generally presenting as a fusiform pulsating expansile tumour behind the right sterno-clavicular articulation. An aneurysm of the transverse arch gives rise to a variety of symptoms, all referable to the pressure of the tumour on the various structures at the root of the neck. Thus it presses on the left recurrent laryngeal nerve. Now, although it is an anatomical fact that all the intrinsic muscles of the larynx are supplied by the recurrent laryngeal nerve, clinically speaking, only the crico-arytenoideus posticus is put out of gear by pressure on the nerve. To explain this, it has been suggested that the fibres which supply that muscle are arranged at the periphery of the nerve, so that it becomes paralysed before the other muscles are affected. The crico-arytenoideus posticus is an abductor of the vocal cord, and the result of its paralysis is over-action of the opposing adductors and the approximation of the cord to the middle line. If both crico-arytenoidei postici are paralysed, as sometimes happens, for instance, in syphilis, the vocal cords may be brought close together, and urgent dyspnœa necessitating tracheotomy may result. In aneurysm of the transverse arch, however, it is only the left one which is paralysed, and the result is a change in the voice and the brassy cough which is so characteristic oi this disorder. Dyspnœa may result from actual pressure of the aneurysm on the trachea, and dysphagia may similarly come from compression of the œsophagus. This explains the necessity, already mentioned, of excluding aneurysm of the transverse arch, before passing an œsophageal bougie in a case of dysphagia. A symptom on which much stress is laid by some is that which is known as tracheal tugging. If, while the patient is in the recumbent position, the larynx is gently but firmly drawn upwards towards the chin, it is said that it can be felt to be pulled down with each beat of the heart, the explanation being that each pulsation in the aneurysm pushes down the left bronchus. Too much reliance should not be placed on the presence or absence of this sign. Œdema of the left side of the face and neck may be caused by pressure of the aneurysm on the left innominate vein.

None of the great arterial trunks of the head and neck is immune from aneurysm, but the diagnosis should not be difficult if the cardinal symptoms are borne in mind. These are a few of the conditions, however, which may possibly lead to confusion—(*i*) a tuberculous abscess situated in the line of an artery, (*ii*) a subclavian artery passing over a supernumerary cervical rib, (*iii*) a pulsating sarcoma, and (*iv*) a goitre.

### Arterio-venous Communications.

These are generally the result of an injury, which damages the walls of an artery and vein lying in juxtaposition, so that the arterial blood is poured into the vein below the lesion. Two varieties are described (*a*) aneurysmal varix in which the artery communicates with the vein direct, and (*b*) varicose aneurysm, in which the vein communicates with a localised dilatation of the artery. Such a distinction does not, as a matter of fact, serve any useful end, since to all intents and purposes the conditions are identical. The result of this, if it occurs in a limb, is the development of an irregular tumour, over which there is a thrill and a bruit, which has been compared to the noise made by a bee in a bottle; after a time the vein below the communication becomes thick and tortuous, and all its radicles are prominent. In long-standing cases there may be hypertrophy of the limb owing to its increased blood supply. In such a case the prognosis is not hopeful, and it has been said that the best thing to do is to leave the condition alone. If any operation be undertaken, the most promising is to tie the artery above and the vein below the communication. Such a communication is not uncommon between the internal carotid artery, and the cavernous sinus after fractures of the anterior fossa of the skull. It causes exophthalmos, with pulsation of the globe of the eye and œdema of the lids and corneo-scleral junction owing to obstruction to the return of venous blood. There may also be damage to the oculomotor nerves leading to an ophthalmoplegia, which is, however, generally transient. The only treatment which holds out any hope of success is ligature of the internal carotid on the affected side. But failure is common on account of the free anastomosis between the arterial trunks at the base of the brain.

